# COVID-19 prevalence and infection control measures at homeless shelters and hostels in high-income countries: a scoping review

**DOI:** 10.1186/s13643-022-02089-x

**Published:** 2022-10-15

**Authors:** Justine Levesque, Jordan Babando, Nathaniel Loranger, Shantel Johnson, David Pugh

**Affiliations:** 1 The Canadian Observatory On Homelessness 6Th Floor Kaneff Tower, York University, 4700 Keele St, Toronto, ON M3J 1P3 Canada; 2 Institute of Criminology and Criminal Justice, Carleton University, Ottawa, ON K1S 5B6 Canada; 3 York University School of Social Work, S880 Ross Building, 4700 Keele St, Toronto, ON M3J1P3 Canada

**Keywords:** COVID-19, Homeless shelters, Shelter workers, Infection control, Prevention

## Abstract

**Background:**

The COVID-19 pandemic has disproportionately impacted homeless populations and service workers, especially within homeless shelter/hostel settings. To date, there have been few evidence syntheses examining outbreaks of COVID-19 among both homeless shelter residents and service workers and no critical review of infection control and prevention (IPAC) measures. This scoping review offers a much-needed synthesis of COVID-19 prevalence within homeless shelters and a review of pertinent IPAC measures.

**Methods:**

We conducted a scoping review that aimed to synthesize academic and gray literature published from March 2020 to July 2021 pertaining to (1) the prevalence of COVID-19 among both residents and staff in homeless shelters and hostels in high-income countries and (2) COVID-19 IPAC strategies applied in these settings. Two reviewers independently screened the literature from several databases that included MEDLINE, PsycInfo, and the WHO’s COVID-19 Global Health Portal. The extracted data was mapped, categorized, and thematically discussed.

**Results:**

Thematic analysis of 77 academic and gray literature documents revealed four key themes: (1) the demographics of COVID-19 in homeless shelters, (2) asymptomatic spread, (3) pre-existing vulnerability of people experiencing homelessness and shelters, and (4) the inconsistency and ineffectiveness of IPAC implementation.

**Conclusion:**

This review offers a useful glimpse into the landscape of COVID-19 outbreaks in homeless shelters/hostels and the major contributing factors to these events. This review revealed that there is no clear indication of generally accepted IPAC standards for shelter residents and workers. This review also illustrated a great need for future research to establish IPAC best practices specifically for homeless shelter/hostel contexts. Finally, the findings from this review reaffirm that homelessness prevention is key to limiting disease outbreaks and the associated negative health outcomes in shelter populations. Limitations of this review included the temporal and database constraints of the search strategy, the exclusion of quality assessments of the literature, and the absence of investigation on the influence of emerging variants on public health policy.

**Systematic review registration:**

This scoping review has not been registered on any database; the protocol is available on York University’s Institutional Repository https://dx.doi.org/10.25071/10315/38513.

**Supplementary Information:**

The online version contains supplementary material available at 10.1186/s13643-022-02089-x.

## Background

As of June 2021, over 539 million cases of the SARS-CoV-2 virus, more commonly known as COVID-19, have been recorded worldwide, thus ensuring that we are bearing witness to a “once-in-a-lifetime pandemic” [1, 2]. This global public health crisis has also intersected with the existing global crisis of homelessness [3].

While homelessness is prevalent worldwide, this review specifically focused on the impacts of COVID-19 among homeless shelter populations in high-income countries. The lack of welfare and social support systems in low-income countries [4] translates to an underdeveloped homeless shelter system as compared to those in high-income countries, complicating the comparison of data from these contexts in this review. Accordingly, literature is more abundant from public health authorities directing shelter systems in high-income countries on interventions for COVID-19 to protect their residents throughout the pandemic which is a central interest of this review.

Given the focus on high-income countries in this review, the authors used the Canadian Observatory on Homelessness’ definition and typology for homelessness [5]. The COH defines homelessness as, “…a situation where an individual, family, or community is without stable, permanent, appropriate housing, and lacks the means to immediately obtain it” [6].

Within the typology of homelessness, unsheltered/absolute homelessness refers to those living on the streets or areas not meant for human habitation, emergency sheltered homelessness refers to those staying in homeless shelters (e.g., overnight or family violence shelters), and; provisionally accommodated homelessness refers to those with temporary accommodations lacking longevity or security (e.g., couch surfing) [5].

People experiencing homelessness (PEH) are disproportionately affected by COVID-19 due to pre-existing medical conditions, inadequate access to preventative health care, and a lack of safe housing options [3, 7]. Point prevalence rates among people experiencing sheltered homelessness (PESH) in Atlanta, Georgia, from April to May 2020 were four times higher than among people experiencing unsheltered homelessness [8]. The severity of the COVID-19 virus is also heightened among PEH as the need for critical care from COVID-19 increases ten-fold for PEH compared to the general population [9].

The risk of contracting COVID-19 increases for PEH in shelters/hostels as living conditions in these settings are often overcrowded and lack stringent public health measures [10]. Outbreaks of COVID-19 have been recorded in homeless shelters and hostels across high-income countries with prevalence rates reaching up to 67% among shelter residents and 30% among shelter staff [8, 11–14, –16].

Some of the widely recommended infection, prevention, and control (IPAC) measures for COVID-19 in shelters throughout the pandemic included physical distancing, isolation and quarantine, symptom screening, environmental cleaning, and testing [17, 18]. Nonetheless, outbreaks have continued, resulting in a substantial amount of literature relating to the prevalence and infection control of COVID-19 in shelters/hostels.

Consequently, there is a great need to synthesize the available evidence on the spread of COVID-19 and the implementation of IPAC measures to enhance the capacity of shelters/hostels to alleviate the rapid spread of COVID-19. IPAC strategies identified in this scoping review also hold potential for preventing other airborne infectious disease outbreaks among PEH residing in congregate living spaces.

To the best of our knowledge, this is the first review of its kind. This scoping review synthesizes academic and gray literature published from March 2020 to July 2021 in response to the following research questions:

- (1) What is known about COVID-19 positivity rates among residents of homeless shelters/hostels and the staff serving this population?
- (2) What infection, prevention, and control measures have been implemented by, or recommended for, homeless shelters/hostels to prevent and mitigate COVID-19 outbreaks?

A handful of evidence syntheses and numerous single studies have explored similar topics, all of which focus on outbreaks during the first wave of the pandemic [19, 20]. This review builds on this existing research while generating new knowledge as we sought to answer our two research questions.

## Methods

### Protocol

A scoping review was conducted following Arksey and O’Malley’s [21] scoping review framework and the subsequently proposed enhancements from Levac et al. [22] and Peters et al. [23]. The search protocol was informed by the guidelines of the Preferred Reporting Items for Systematic Reviews and Meta-Analyses extension for scoping reviews (PRISMA-ScR) [24]. See Additional file 1 for the updated PRISMA 2020 Checklist. Academic and gray literature published between March 2020 and July 2021 were included in this review. The protocol for this scoping review is available as a preprint on York University’s Institutional Repository (https://dx.doi.org/10.25071/10315/38513). This review was completed in five stages: (1) identifying the research question, (2) identifying relevant studies, (3) study selection, (4) charting the data, and (5) collating, summarizing, and reporting the results [21].

### Search strategy

An initial limited search of two databases (MEDLINE and PsycINFO via OVID) was conducted following consultations with a research librarian at York University to refine the search strategy. An extensive search for academic and gray literature was conducted between June 7 and August 6, 2021. Nine databases were searched for academic literature including MEDLINE and PsycInfo via OVID, Scopus, CINAHL, Social Sciences Abstracts, medRxiv, ProQuest, Google Scholar, and the WHO’s COVID-19 Global Health Portal. Academic databases were last searched on June 23, 2021. A search for relevant gray literature was then conducted using the following databases and webpages: United Nations Database, OpenGrey, WorldCat, CADTH COVID-19 Grey Literature Resources, Dahdaleh COVID-19 portal, Canadian and European Observatories on Homelessness publications, Centers for Disease Control and Prevention (CDC), National Health Care for the Homeless Council (NHCHC), Public Health Agency of Canada (PHAC), Canadian Network for the Health and Housing of People Experiencing Homelessness (CNH3), and Healthy London. Gray literature repositories were last searched on July 21, 2021. Concurrently, while searching for literature, the review team contacted experts and researchers in the field to identify additional relevant literature. The search strategy concluded with a scan of the reference lists of the documents included in the full-text review stage (See Additional file 2 for documents scanned for references). Reference list scanning was completed on August 6, 2021, and the remaining articles identified by the reference checks were extracted from databases on August 6, 2021.

The following terms were used to search all databases, repositories, and webpages, organized by population, concept, and context of interest:

- (“Homeless persons” OR “Homeless people” OR “Homeless youth” OR “Homeless adults” OR “People experiencing homelessness” OR “Homelessness”).
- AND
- (“COVID-19” OR “SARS-CoV-2” OR “Coronavirus” OR “Coronavirus Disease” OR “Coronavirus Disease 2019”).
- AND
- (“Homeless shelter” OR “Emergency shelter” OR “Homeless hostel” OR “Hostel” OR “Warming center” OR “Family violence shelter”).

Terms were used either individually or in combination across the databases. Date filters/limits were used when available to restrict articles/documents to those released from 2020 and onward. A full electronic strategy for MEDLINE is provided in Additional file 3. Using this search strategy, 1390 documents were identified and uploaded to Covidence, a systematic review management platform.

### Inclusion and exclusion criteria

The population, concept, and context (PCC) framework informed the eligibility criteria for this scoping review [23]. The populations of interest were PESH and staff at homeless shelters and/or hostels. No age restrictions were applied to the populations of interest. The concepts investigated in this review included point and/or period prevalence as indicated by COVID-19 test positivity rates. Point prevalence refers to the proportion of a population with the condition of interest at a specific point in time such as one specific date or span of weeks, whereas period prevalence is the proportion of a population with the condition of interest during a specific time period (e.g., 12 months) [25]. Point and period prevalence were both included in this review given that the time period of testing events for COVID-19 varied by location (e.g., single dates, weeks, or months). The other concepts of interest in this review were the infection control measures implemented or recommended to prevent or slow the spread of airborne and respiratory diseases. For COVID-19, this included hand and respiratory etiquette, physical distancing, screening, and testing, environmental cleaning, and isolation or quarantine.

Academic literature eligible for inclusion in this review included research studies, commentaries, preprints, dissertations, and theses, while gray literature included organization documents, government documents, and policy papers. Newspaper articles, press releases, and magazine articles were excluded. All documents needed to be written in English to be included. Literature that reported COVID-19 data among people experiencing unsheltered, hidden, or provisionally accommodated homelessness or outbreaks exclusively in other homeless service sites (e.g., encampments) were excluded. While outbreaks of COVID-19 among other homeless populations is an important topic of research, it is beyond the scope of this review given that implementation and evaluation of IPAC measures are more variable among unsheltered homeless populations as they may not be required to follow such measures when residing in encampments, sleeping outside, or couch surfing. Literature from middle and low-income countries, as measured by the World Bank [24], was excluded. Studies and documents that discussed other epidemiological measures of COVID-19 (e.g., standardized mortality rate) or epidemiological and infection control measures for diseases other than COVID-19 (e.g., influenza) were excluded.

### Article selection

**Fig. 1:**
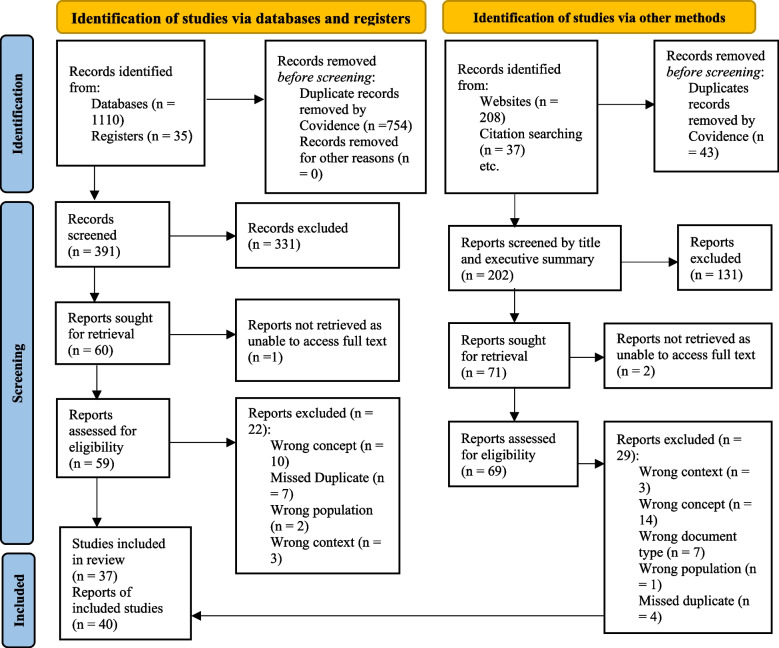
PRISMA 2020 flow diagram of article selection and review process

All reviewers piloted the inclusion and exclusion criteria to ensure there was a shared understanding. Using Covidence, two reviewers (NL and SJ) independently screened titles and abstracts/executive summaries, and then full texts, consulting with a third reviewer (JL) to resolve disagreements. Articles were excluded when they were the wrong document types, conducted in the wrong context/setting or population, investigated the wrong concept, or unavailable as full text. For example, a study from Jadidzadeh and Kneebone [26] satisfies some inclusion criteria as they investigated shelter use patterns among PESH and the implications for the spread of the COVID-19 virus. While this article focused on the correct population and shelters in a high-income country, it did not report the prevalence of COVID-19 among PESH or specific IPAC measures utilized in the shelter and was therefore excluded from the review. Most duplicates were automatically removed through Covidence, with the remaining duplicates manually removed by the reviewers when necessary. A total of 77 documents were included in this analysis. A PRISMA flow diagram is presented in Fig. 1 in accordance with the PRISMA 2020 statement to demonstrate the searching and screening process utilized in this review [27].

### Data extraction

A data extraction tool was developed using the sample extraction instrument provided by Peters et al. [23] and converted to an Excel spreadsheet. The data extraction tool is attached as Additional file 4. All reviewers piloted the data extraction tool to ensure that data extraction was consistent. Four reviewers (JL, NL, SJ, and DP) extracted data independently. The data extracted included author(s), date of publication, document type, the population of interest (PESH or staff), context (shelter/hostel/both), location (city, province/state, and country), type of environment (e.g., urban, rural, remote), participant demographics, testing date(s), the number of testing sites, COVID-19 positivity rate among PESH and staff, and instructions for specific infection, prevention, and control strategies (e.g., screening, testing, physical distancing, and food safety). See Additional file 5 for a condensed version of the data repository from all 77 documents and Table 1 for a summary of key data extracted from all documents.

**Table 1:** Summary of key data extracted from documents

Author(s)	Date	Participants	Country	COVID-19 test positivity			IPAC measure(s)
Aranda-Díaz et al. [28]	2021	Shelter residents and staff	USA	Residents 10/393 = 2.5%		Staff 4/232 = 1.7%	Testing Isolation & quarantine
Arroyo [29]	2020	N/A	USA	N/A			Screening & surveillance Testing Hand & respiratory etiquette Physical distancing Isolation & quarantine PPE
Baggett et al. [30]	2020	Shelter residents	USA	Residents 147/408 = 36%		N/A	N/A
Baggett et al. [12]	2020	Shelter residents	USA	Residents 147/408 = 36%		N/A	N/A
Baggett et al. [31]	2020	Shelter residents	USA	Residents & marginally Housed 429/1297 = 33.1%			Screening & surveillance Testing Cleaning & waste management Physical distancing PPE Other
Baggett et al. [32]	2020	N/A	USA	N/A			Screening & surveillance Testing Physical distancing Other
Baltimore City Health Department [33]	2020	N/A	USA	N/A			Screening & Surveillance Testing
Baral et al. [34]	2020	N/A	USA	N/A			Screening & surveillance Testing Hand & respiratory etiquette Cleaning & waste management Physical distancing Isolation & quarantine Food safety PPE Other
Barocas et al. [35]	2021	N/A	USA	N/A			Screening & surveillance PPE
Bond [36]	2020	N/A	USA	N/A			Screening & surveillance Testing Isolation & quarantine
California Business Consumer Services and Housing Agency [37]	2020	N/A	USA	N/A			Screening & surveillance Hand & respiratory etiquette Cleaning & waste management Physical distancing Isolation & quarantine Food safety PPE
CNH3 [38]	2020	N/A	Canada	N/A			Isolation & quarantine PPE
Center on Budget and Policy Priorities et al. [39]	2020	N/A	USA	N/A			Screening & surveillance Testing Cleaning & waste management Physical distancing Isolation & quarantine Food safety PPE Other
CDC [18]	2020	N/A	USA	N/A			Screening & surveillance Hand & respiratory etiquette Cleaning & waste management Physical distancing Isolation & quarantine PPE
CDC [40]	2020	N/A	USA	N/A			Screening & surveillance Testing Hand & respiratory etiquette Cleaning & waste management Physical distancing Isolation & quarantine Food safety PPE Other
CDC [41]	2021	N/A	USA	N/A			Screening & surveillance Testing Isolation & quarantine
Chapman et al. [17]	2021	Shelter residents and staff	USA	N/A			Screening & surveillance Testing Isolation & quarantine PPE
Chicago Homelessness and Health Response Group For Equity [42]	2020	Shelter residents	USA	Residents 558/2146 = 26%	N/A		Screening & surveillance Testing Hand & respiratory etiquette Physical distancing Isolation & quarantine Food safety PPE
Department of Homeless Services [43]	2020	N/A	USA	N/A			Screening & surveillance Cleaning & waste management Physical distancing Isolation & quarantine Food safety Other
Department of Homeless Services [44]	2020	N/A	USA	N/A			Physical distancing Food safety Other
Department of Homeless Services [45]	2020	N/A	USA	N/A			Screening & surveillance Cleaning & waste management Physical distancing Isolation & quarantine PPE Other
Department of Public Health [46]	2021	N/A	USA	N/A			Screening & surveillance Hand & respiratory etiquette Cleaning & waste management Physical distancing Isolation & quarantine Food safety PPE Other
Falvo [47]	2020	N/A	Canada	N/A			Screening & surveillance Physical distancing Isolation & quarantine PPE Other
Gewirtz O’Brien et al. [48]	2021	N/A	Multi-country	N/A			Testing Physical distancing Isolation & quarantine PPE Other
Ghinai et al. [49]	2020	Shelter residents and staff	USA	N/A			Screening & surveillance Testing Physical distancing Other
Government of New Brunswick [50]	2020	N/A	Canada	N/A			Screening & surveillance Hand & respiratory etiquette Cleaning & waste management Physical distancing Isolation & quarantine Food safety PPE
Government of New Brunswick [51]	2021	N/A	Canada	N/A			Screening & surveillance Hand & respiratory etiquette Cleaning & waste management Physical distancing Isolation & quarantine Food safety PPE Other
Government of Northwest Territories [52]	2020	N/A	Canada	N/A			Screening & surveillance Hand & respiratory etiquette Cleaning & waste management Physical distancing Isolation & quarantine Food safety PPE
Government of Yukon [53]	2020	N/A	Canada	N/A			Screening & surveillance Cleaning & waste management Physical distancing Isolation & quarantine
Imbert et al. [8]	2020	Shelter residents and staff	USA	Residents 101/150 = 67%	Staff 10/60 = 17%		N/A
Jameson [54]	2021	Shelter residents and staff	USA	Residents and Staff Combined Prevalence Testing Window A: 69/251 = 27.4% Testing Window B: 20/61 = 32.7% Testing Window C: 2/188 = 1.1% Testing Window D: 1/167 = 0.6%			Screening & surveillance Testing Cleaning & waste management Isolation & quarantine
Karb et al. [55]	2020	Shelter residents	USA	Residents 35/399 = 11.7%	Staff N/A		Screening & surveillance Physical distancing Food safety PPE Other
Kelly et al. [56]	2020	N/A	USA	N/A			Screening & surveillance Physical distancing Isolation & quarantine PPE
Kiran et al. [11]	2021	Shelter residents	Canada	Residents 69/504 = 13.7% and 11/496 = 2.2%	Staff N/A		N/A
Kiran et al. [57]	2020	Shelter residents	Canada	Residents 69/504 = 13.7% and 11/496 = 2.2%	Staff N/A		N/A
Lewer et al. [14]	2020	Shelter residents	UK	Incidence is reported Scenario A: 1888/46565 = 4.1%, Scenario B: 22,933/46565 = 49.3%, Scenario C: 1025/46565 = 6.3%, Scenario D: 12,151/46565 = 30.1%. Scenario E: 8497/46565 = 22.4%. Scenario F: 1754/46565 = 7.8%. Scenario G: 13,320/46565 = 32.7%, Scenario H: 9946/46565 = 25.4%			Testing Hand & respiratory etiquette Physical distancing Isolation & quarantine
Lindner et al. [58]	2020	Shelter residents	Germany	Residents 0/118 = 0%	Staff N/A		Testing
London Coronavirus Response Cell [59]	2020	N/A	UK	N/A			Screening & surveillance Testing Hand & respiratory etiquette Cleaning & waste management Physical distancing Isolation & quarantine Food safety PPE Other
Loubière et al. [10]	2021	Shelter residents	France	Residents 65/1156 = 5.6%	Staff N/A		N/A
Ly et al. [16]	2021	Shelter residents	France	Residents 26/308 = 8.4%	Staff N/A		N/A
Ly et al. [60]	2021	Shelter residents and staff	France	Residents: 37/441 = 9.0%	Staff N/A		N/A
Mercy Care [61]	2020	N/A	USA	N/A			Testing Hand & respiratory etiquette Cleaning & waste management Physical distancing PPE
Ministry of Health [62]	2020	N/A	Canada	N/A			Screening & surveillance Hand & respiratory etiquette Cleaning & waste management Physical distancing Isolation & quarantine Food safety PPE
Mohsenpour et al. [19]	2021	Shelter residents and staff	Multi-country	N/A			N/A
Monroe County Shelter Bed Task Group [63]	2020	N/A	USA	N/A			Screening & surveillance Hand & respiratory etiquette Cleaning & waste management Physical distancing Isolation & quarantine Food safety PPE
Mosites et al. [15]	2020	Shelter residents and staff	USA	Residents Seattle (shelters 1–3) = 31/79 (17%) Boston = 147/408 (36%) San Francisco = 95/143 (66%) Atlanta = 10/249 (4%)	Staff Seattle (shelters 1–3) = 6/35 (17%) Seattle shelters 4–15) = 1/106 (1%) Boston = 15/50 (30%) San Francisco = 10/63 (16%) Atlanta 1/59 (2%)		N/A
Mosquera-Bruno [64]	2020	N/A	USA	N/A			Physical distancing Food safety PPE Other
Murray [65]	2021	N/A	Canada	N/A			Screening & surveillance Testing Cleaning & waste management Physical distancing Isolation & quarantine Food safety PPE Other
National Alliance to End Homelessness [66]	2020	N/A	USA	N/A			Screening & surveillance Isolation & quarantine Other
National Collaborating Center for Methods and Tools [67]	2020	Shelter residents	Multi-country	Residents Samuels—35/299 = 11.7% Baggett -147/408 = 36.0%) Bodkin—1/ 104 = 1.0% Tobolowsky—31/195 = 18.9%	Staff Bodkin—7/141 = 5.0% Tobolowsky—6/38 = 15.8%		Screening & surveillance Testing Cleaning & waste management Physical distancing Isolation & quarantine PPE
National Health Care for the Homeless Council [68]	2020	N/A	USA	N/A			Screening & surveillance Physical distancing Isolation & quarantine Food safety
North Carolina Department of Health and Human Services [69]	2020	N/A	USA	N/A			Hand & respiratory etiquette Cleaning & waste management Physical distancing Isolation & quarantine Food safety PPE Other
NYC Department of Health and Mental Hygiene [70]	2020	N/A	USA	N/A			Screening & surveillance Hand & respiratory etiquette Cleaning & waste management Physical distancing Isolation & quarantine Food safety
O’Shea et al. [71]	2021	Shelter Residents And Staff	Canada	Residents 1/104 = 1.0%	Staff 7/141 = 5.0%		Screening & surveillance Testing Physical distancing Isolation & quarantine
Office of Temporary and Disability Assistance [72]	2020	N/A	USA	N/A			Screening & surveillance Hand & respiratory etiquette Cleaning & waste management Physical distancing Food safety PPE Other
Public Health Agency of Canada [73]	20,202	N/A	Canada	N/A			Screening & surveillance Hand & respiratory etiquette Cleaning & waste management Physical distancing Isolation & quarantine PPE Other
Public Health England [74]	2021	N/A	UK	N/A			Screening & surveillance Hand & respiratory etiquette Cleaning & waste management Physical distancing Isolation & quarantine PPE Other
Public Health Ontario [75]	2021	N/A	Canada	N/A			Screening & surveillance Hand & respiratory etiquette Physical distancing Isolation & quarantine Food safety Other
Ralli et al. [76]	2021	N/A	Multi-country	N/A			Screening & surveillance Testing Hand & respiratory etiquette Physical distancing PPE Other
Ralli et al. [77]	2021	Shelter residents and staff	Italy	Not specified = 2.0%			Screening & surveillance Testing Hand & respiratory etiquette PPE
Ralli et al. [13]	2021	Shelter residents and staff	Italy	Residents and staff combined 12/298 = 4%			N/A
Rao et al. [78]	2021	Staff	USA	Residents N/A	Staff 16/106 = 15.09%		Testing Hand & respiratory etiquette Cleaning & waste management Physical distancing Ppe
Redditt et al. [79]	2020	Shelter residents	Canada	Residents 25/60 = 41.7%	Staff N/A		Screening & surveillance Testing Isolation & quarantine
Roederer et al. [80]	2021	Shelter residents	France	Residents 426/818 = 52%	Staff N/A		N/A
Rogers et al. [81]	2021	Shelter residents and staff	USA	Residents 25/1275 = 1.96%	Staff 4/159 = 2.51%		N/A
Roland et al. [82]	2021	Shelter residents	Belgium	Residents 91/1994 = 4.6%	Staff N/A		N/A
Samuels et al. [83]	2020	Shelter residents	USA	Residents 35/299 = 11.7%	Staff N/A		Screening & surveillance Testing Physical distancing Food safety Ppe Other
Self et al. [84]	2021	Shelter residents and staff	USA	Residents and staff combined Median prevalence by facility was 2.9% (range = 0–71.4%)			Screening & surveillance Physical distancing Other
Tobolowsky et al. [85]	2020	Shelter residents and staff	USA	Residents 35/195 = 18%	Staff 8/38 = 21%		Testing Hand & respiratory etiquette Physical distancing Ppe
Udechukwu et al. [86]	2021	N/A	Multi-country	N/A			Testing Hand & respiratory etiquette
Vancouver Coastal Health [87]	2020	N/A	Canada	N/A			Screening & surveillance Hand & respiratory etiquette Cleaning & waste management Physical distancing Isolation & quarantine Food safety PPE Other
Wang et al. [88]	2020	Shelter residents	Canada	Residents 372/10588 = 3.5%	Staff N/A		N/A
Wang et al. [89]	2020	N/A	Multi-country	N/A			Testing Physical distancing
Yoon et al. [90]	2020	Shelter residents	USA	Residents 36.1684 = 2.1%	Staff N/A		Screening & surveillance Testing Physical distancing

### Data synthesis

Descriptive statistics were produced related to the characteristics of the literature, geographic distribution, and concepts of interest (e.g., prevalence and infection control) for this scoping review. All reviewers tracked preliminary patterns observed while extracting data. Fourteen preliminary patterns were identified and organized in relation to the prevalence and/or infection control. Documents were grouped in the condensed data repository according to the concept that was central to each document (e.g., prevalence, infection control, or both). Three reviewers (JL, JB, and NL) conducted further thematic analysis of the data extracted from the 77 documents and collapsed the 14 preliminary patterns into four overarching themes.

## Results

### Quantitative

In total, 77 of the 1390 identified documents met the selection criteria of this review. Among these, 37 (48%) were peer-reviewed literature including commentaries and research papers, and 40 (52%) were gray literature documents such as government documents and policy papers (Fig. 1). Nearly two thirds (62.3%) of these documents were published in 2020 with most studies published in April 2020, and 37.7% in 2021.

**Fig. 2:**
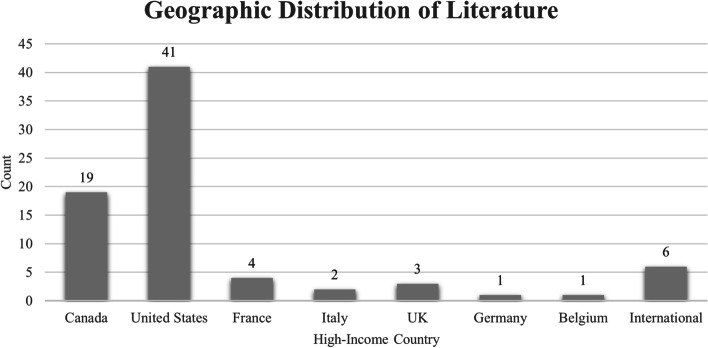
Distribution of literature by country

More than half of the documents in this review (53.2%) were based in the USA followed by Canada (24.7%), France (5.2%), the United Kingdom (3.9%), Italy (2.6%), Germany (1.3%), and Belgium (1.3%). Six (7.8%) of the documents contained information from multiple countries and were classified as multi-country documents (Fig. 2). In total, 44 (57.1%) documents in this review reported infection control-focused data, 17 (22.1%) reported prevalence-focused data, and 16 (20.8%) reported information on both prevalence rates and infection control measures in shelters/hostels. The majority (62.3%) of the documents considered both residents and staff at homeless shelters/hostels while 28 (36.5%) focused solely on residents at shelters/hostels and one (1.2%) solely on staff at shelters/hostels.

The reported COVID-19 positivity rates among residents of homeless shelters/hostels ranged from 0 to 67% [8, 15, 19, 30, 58, 80]. The lowest positivity rate of 0% was recorded among shelter residents in Germany in July 2020 [58], and the highest positivity rate of 67% was recorded among shelter residents in the USA during April 2020 [8]. Similarly, COVID-19 positivity rates among staff ranged from 1 to 30% [8, 15, 19, 71, 78, 81, 85]. The lowest and highest positivity rates among staff were reported in the same study by Mosites et al. [15] from testing events during March and April 2020 in the USA. Three studies did not separate testing results of residents and staff and reported overall shelter positivity rates ranging from 0 to 71.4% in the USA, and 4% in Italy [13, 54, 84] between April and September 2020.

#### Infection control measures

We collapsed the IPAC measures into nine categories across the 77 documents: (a) screening and surveillance, (b) testing, (c) hand and respiratory etiquette (e.g., hand washing and masking), (d) personal protective equipment (PPE), (e) environmental cleaning and waste management, (f) physical distancing, (g) isolation and quarantine, (h) food safety, and (i) other (e.g., vaccinations, enhanced ventilation, and visitor restrictions). These nine categories were selected as they were commonly used across the articles included in the review. Furthermore, these terms are standard IPAC terms used before the pandemic in different settings (e.g., shelters, hospitals, long-term care, childcare) to prevent the spread of airborne illnesses. See Additional file 6 for definitions of the IPAC measures.

**Fig. 3:**
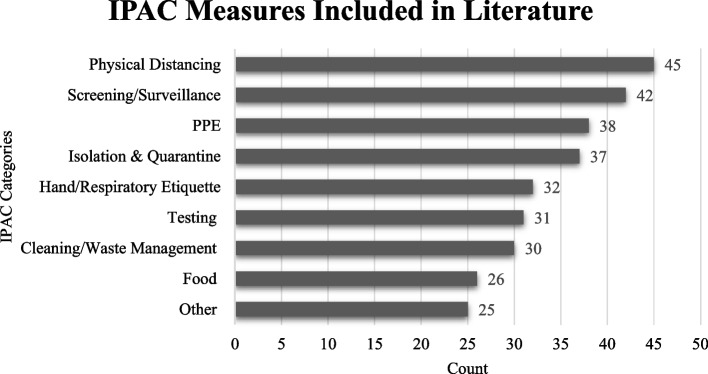
IPAC measures discussed in scoping review documents

While many of the IPAC measures appeared frequently in the literature of this scoping review, no individual IPAC measure appeared in every single document (Fig. 3). Physical distancing was the most common IPAC measure appearing in 45 (57.4%) of the documents [29, 37, 40, 43, 44, 44, 91]. Screening and surveillance were the second most common IPAC measures discussed in 42 (54.5%) of the documents [36, 41, 50, 52, 53, 56, 59, 62, 63, 65, 68, 70, 72] (Fig. 3). Guidance for environmental cleaning/waste management and food safety were the least common IPAC measures appearing in 30 (39%) and 26 (33.8%) of the documents, respectively [61, 64, 69, 86, 87, 92, 93].

#### Qualitative thematic findings

##### Theme #1: Profiling COVID-19 in homeless shelters

In total, 25 documents reported demographic information for participants, and of these 25, 18 specifically reported the socio-demographics of participants who tested positive for COVID-19. In this sample of existing research, we observed that older adults aged 50 and above, those identifying as male, and identifying as People of Color were overrepresented among cohorts testing positive for COVID-19.

**Age**

Broader homeless populations and PESH in high-income countries often include more youth and adults under age 50 than were identified in study populations in this review [94, 95, –97]. For example, recent data from the UK indicated that only 8% of the people experiencing homelessness were aged 55 and older with the largest proportion (32%) being aged 35–44 [96, 97]. Likewise, in Canada, youth under the age of 24 represented 18.7% of PEH [94]. Of studies reporting mean/median age [11, 30, 31, 57, 81, 85, 90], only one reported a mean/median age among COVID-19-positive participants below age 40 [79]. In some cases, the mean/median age exceeded 50 [30, 81, 85]. Importantly, large proportions of participants in older adult age groups (50–64 and 65 +) were recorded [30, 81, 85]. In Toronto and Marseille respectively, 47.5% and 18.9% of shelter residents who tested posted for COVID-19 were ages 50 and older [11, 60]. Older adults experiencing sheltered homelessness appear to be a particularly vulnerable group during the pandemic.

**Sex/gender**

Certain studies reported sex, while others reported gender. Nonetheless, it was clear that males represented the majority of shelter residents testing positive for COVID-19. Available data demonstrates that men outnumber women in the shelter systems in high-income countries [94, 98]. Sex and/or gender of COVID-19-positive shelter residents were reported in 12 studies and in every one of these studies 65% or more of the shelter residents that tested positive for COVID-19 identified as male [10, 11, 30, 31, 49, 57, 60, 79, 81–83, 85]. During outbreaks at shelters in Boston, King County, and Toronto, this proportion increased to over 80% of positive cases [30, 79, 81]. While men occupy a larger proportion of the shelter system, women still represent a significant proportion of PEH and shelter residents than is reflected in the populations of shelter residents testing positive for COVID-19. In Canada, 27.3% of PEH were women and 12% of shelter beds in Canada are allocated to women specifically [94, 99]. Similarly, nearly one third (31.4%) of shelter residents identified as female in the USA [98]. The data from this review highlighted a relationship between sheltered homelessness, sex/gender, and COVID-19. However, given that women are more likely to experience hidden homelessness [100], and may be missed in the shelter system, it is difficult to assess the extent to which COVID-19 positivity is influenced by gender/sex among PESH.

**Race**

While there are variations in the types of racial/ethnic categories recorded in individual documents, it is still evident that racialized groups are overrepresented among cohorts of shelter residents testing positive for COVID-19. Available data indicates that in high-income countries, shelter and homeless populations are comprised of a majority of White/Caucasian individuals [94, 96–98]. In the UK, in 2018–2019 over 60% of individuals experiencing homelessness identified as White/Caucasian and 50% of PESH in the USA identified as White/Caucasian [97, 98]. However, in Canada individuals identifying as Indigenous (First Nations, Metis, and Inuit) represented 28–34% of shelter populations [94]. While White/Caucasian populations make up a majority of shelter and homeless populations, there were high prevalence rates of COVID-19 recorded among racialized groups in shelters across the studies included in this review. In Chicago, 63.8% of shelter residents with a positive test result identified as non-Hispanic Black [49]; in Boston, 31.9% of COVID-19-positive shelter residents identified as Black/African American and 16.1% as Hispanic/Latino, and in Toronto, 61.9% identified as racialized including Black, Asian, and Latino [11]. Racialized communities experience homelessness at higher rates than White/Caucasian individuals, and these racial disparities are seen again among shelter residents testing positive for COVID-19.

##### Theme #2: Silently sick: asymptomatic spread

The cohorts of homeless shelter residents and staff testing positive for COVID-19 throughout 2020 and 2021 were also characterized by asymptomatic carriers of the virus. Asymptomatic spread is a distinguishing feature of the COVID-19 virus contributing to its ability to rapidly infect scores of vulnerable populations. One in five documents included in this literature review discussed the asymptomatic spread of the virus among homeless shelter/hostel residents and staff within these congregate settings [8, 11–13, 19, 28, 30, 42, 57, 60, 67, 77, 79, 82, 83]. Ralli et al. [13] reported that 75% of positive cases from staff and residents at homeless shelters in Italy were asymptomatic when tested and in the 14 days leading up to testing. Ralli et al. [13] emphasized the critical point that the lack of symptoms among asymptomatic individuals does not translate into a lack of harm with many studies demonstrating lung abnormalities among asymptomatic cases. Most studies in this review focused on the importance of identifying asymptomatic carriers in homeless shelters because of their significant role in disease transmission [60, 82, 83]. However, one study commented on the pre-existing health vulnerabilities and challenges accessing healthcare that PESH face which could exacerbate the health impacts they incur from COVID-19 [13]. In the USA and Belgium, 87.8% and 93% of positive cases from shelter testing events were asymptomatic at the time of diagnosis [12, 82]. While shelter/hostel residents and staff may lack symptoms of COVID-19, the infectivity of the virus is not diminished allowing the virus to spread undetected [13]. Given that infectivity and physiological damages are not reduced in the absence of symptoms, many authors of the studies included in this review called for universal testing, more stringent hygiene, and physical distancing measures in shelters [13, 57, 76, 77, 82, 83]. Moreover, multiple studies concluded that universal testing is not an inadequate strategy to prevent COVID-19 transmission among shelter residents and staff given that many cases detected through testing were asymptomatic [79, 82, 83].

##### Theme #3: Pre-existing vulnerability of people experiencing homelessness and shelters

Regardless of the type of literature analyzed (e.g., research article, policy paper, and government document), there was recognition that PEH are disproportionately impacted by the COVID-19 pandemic. The explanations consistently provided in the literature for these inequitable, yet preventable, impacts were as follows: (a) pre-existing medical conditions and barriers to accessing healthcare, (b) overcrowding and unsanitary living conditions in shelters/hostels, and (c) limited access to physical materials to protect against the virus (e.g., hand sanitizer and personal protective equipment) [51, 75, 90]. As early as March 2020, the National Alliance to End Homelessness released a document citing both medical concerns and shelter capacity as threats to PEH as the virus began to spread [66]. Among other recommendations, this organization called for mitigation of outbreaks among shelter populations by extending Medicaid in the USA to homeless adults and expanding shelter capacities and operating hours [66]—a trend seen across many other documents in this review [34, 39, 42, 47].

Nonetheless, a pattern of higher positivity rates within shelters compared to the general population during the same time periods was observed in the available data [11, 80]. One rapid review reported that the prevalence of COVID-19 was between 2 and 18 times higher in congregate settings including shelters compared to the general population [67]. Similarly, a study of prevalence among frontline staff at homeless shelters in Hamilton, Ontario, revealed they were being infected at higher rates than the general population [71].

##### Theme #4: Inconsistency and effectiveness of IPAC implementation

IPAC measures are part of reopening plans for countries, cities, and organizations worldwide. However, as outbreaks of COVID-19 have continued, many have wondered about the effectiveness of these measures in stopping the spread of COVID-19. The question of effectiveness was also a key theme observed in this review. The short answer to this question is that there is not enough evidence to unequivocally determine the individual or collective utility of IPAC measures in preventing COVID-19 transmission in shelter/hostel settings. However, preliminary research suggests that using individual IPAC measures, such as screening, is not enough to prevent COVID-19 from spreading in shelters/hostels. Rather, utilizing a combination of IPAC measures may best equip these facilities to control the spread of COVID-19 [12, 17, 77].

Three documents [14, 17, 32] modeled outbreaks of COVID-19 in homeless shelters in the presence of different IPAC measures and different incidence rates in the broader community. Chapman et al. reported that a combination of daily symptom screening, twice-weekly testing, universal masking, and removal of high-risk individuals had the best chance of preventing an outbreak [17]. However, given that all IPAC measures have limitations, this combination prevented only a small number of outbreaks even in the lowest risk transmission settings [17]. Baggett et al. [32] reported that a combined effort of daily symptom screening with testing of symptomatic individuals, including bi-weekly universal PCR testing and alternative care sites for mild/moderate cases of COVID-19, substantially reduced infections. While these documents demonstrated some success for a combination of IPAC measures, they also concluded that outbreaks of COVID-19 are still highly probable in these settings and that IPAC measures would do little to prevent outbreaks in cities with high community incidence [17]. Ultimately, closing dormitory-style shelters in England during the first wave prevented mass spreading in these units, but mass closures of homeless shelters/hostels are impractical as they would remove much-needed housing resources from PEH [14, 17].

A combination of IPAC measures demonstrated some potential in preventing COVID-19 transmission in shelter settings, yet in practice, there was inconsistency in the implementation of IPAC measures based on a shelter’s capacity to follow recommended guidelines. Consistency in the implementation of IPAC measures and the specific IPAC measures used may contribute to the overall effectiveness of these measures to prevent COVID-19. We observed an assumption across many of these documents, and in particular the gray literature, that shelters/hostels would not be able to adhere to the recommended IPAC measures. Some organizational and government documents implied and at times explicitly stated that shelters/hostels would not be able to follow guidelines for specific IPAC measures [38, 42, 53, 69]. Conflicting and downgraded measures to deal with the unique circumstances of shelters/hostels have been consistently observed across the literature in this review. For example, in a document from the Los Angeles Department of Public Health, it was recommended that three-foot spacing between beds be used where six-foot spacing is not possible [46]. Similarly, shelters were advised to use different materials such as lockers and curtains to create temporary barriers between shelter residents; however, the effectiveness of such materials to prevent the spread of COVID-19 is unknown [44].

Conflicting modifications of recommended IPAC measures were also observed in these documents. One example of this was seen in one document that advised that the grouping of symptomatic individuals is not optimal [42] whereas two other documents recommended shelters/hostels do this very thing [73, 74]. In two documents from the same organization in New York which both outline COVID-19 best practices for homeless shelters, masking for shelter residents is included as a best practice in one [43] but is omitted in the other [44]. Studies that have attempted to model outbreaks in the presence of different IPAC measures [14, 17, 32] have not accounted for these modified strategies that were utilized in practice by shelters/hostels. Therefore, the question of effectiveness extends to these modified IPAC measures individually and as a combined approach to preventing COVID-19 transmission in shelter/hostel settings. Nonetheless, IPAC measures have been presented as crucial tools to stop the spread of COVID-19, and shelters/hostels have undoubtedly observed the consequences of having a lack of resources to effectively use these tools.

## Discussion

In this scoping review, we bring new insights into the impacts of COVID-19 on PESH and staff in homeless shelters/hostels as our review recorded outbreaks of COVID-19 in shelters/hostels across seven high-income countries over the first 16 months of the pandemic. Through this review, we identified subpopulations that were overrepresented among the cohorts of shelter residents testing positive for COVID-19, specifically adults aged 40 and older, males, and People of Color. This review also revealed gaps in the pandemic/emergency response protocols and capacities of homeless shelter systems. While homeless shelter systems in each country operate differently, the shortcomings in pandemic/emergency response capacities appear to be consistent across the high-income countries from which literature was reviewed, unlike recommended IPAC measures for COVID-19 which are inconsistently recommended and/or utilized.

Across much of the literature in this review, there was an assumption that shelters could not adhere to IPAC measures that were recommended for their settings. Statements in the documents often explicitly instructed homeless shelter/hostel operators to plan according to their inability to accurately follow IPAC guidelines. When shelter systems are not supported to provide safe and sanitary living conditions, the health of PEH who seek refuge in these places will continue to suffer during this pandemic as well as outbreaks of other airborne infectious diseases. This review illustrates the importance of employing an intersectional approach in any research related to PEH in that relationships exist between the risk of contracting COVID-19 and the age, gender, and race of shelter populations. Thus, certain groups of PEH may be differentially impacted as they experience the pandemic and other inadequacies in shelter systems. The authors, therefore, suggest that shelter systems in high-income countries such as Canada, the USA, the UK, and France receive the resources—namely financial—to support permanent and sustainable changes to their facilities and improve the working and living conditions for shelter residents and staff.

Based on the evidence from this review, we recommend that these changes can include physical upgrades to these facilities such as (1) the expansion of physical shelter spaces to ensure suitable distancing for sleeping spaces and isolation/quarantine spaces for ill residents and (2) upgrades to heating, ventilation, and air conditioning (HVAC) systems. These ideas are not new; rather, this review supports what others have called for even before the onset of the COVID-19 pandemic. Moffa et al. [101] recommended similar shelter transformations in the USA in 2019 and the pandemic demonstrated the consequences of delaying such transformations. Similarly, regional emergency response plans tailored to homeless shelters/hostels must also be developed in partnership with federal and provincial/state governments to ensure that facilities are allocated appropriate resources during disease outbreaks to prevent mass transmission among residents and staff. These emergency response plans can capture temporary alterations to shelters/hostels during disease outbreaks that have been tested during the COVID-19 pandemic. These reversible adaptations include the implementation of rapid testing, screening, and surveillance teams, converting designated areas to isolation/quarantine spaces, and accessing stockpiles of PPE which have been effective in preventing transmission in some shelters [71].

Nevertheless, it is important to note the shortcomings of IPAC measures. Early IPAC measures that were applied to congregate living, such as shelters, were originally developed for health care settings; accordingly, such measures are not directly applicable [38]. As Kiran et al. [11] explain, establishing IPAC measures may be difficult because there is still no clear strategy for detecting COVID-19 among homeless populations. One of the typical IPAC measures employed has been routine contract tracing and quarantining. However, the transient nature of PEH does not allow for the same routine contact tracing and quarantining procedures as in the general population [54]. Instead, some documents here have recommended a need to test more widely and ensure isolation of positive cases within homeless shelters [10, 54, 90]. Furthermore, IPAC measures that recommend symptom screening in congregate settings were found to be ineffective, and instead, regular testing of asymptomatic individuals is recommended [11, 13, 57, 60, 83]. Finally, wide-scale testing has been recommended in some IPAC literature, yet it can be hard to administer due to a lack of staffing and laboratory resources [58] and indeed might actually increase the risk of staff contacting COVID-19 [78].

Shelters often lack the space for proper social distancing guidelines that IPAC recommends, and instead, they should enhance non-congregated settings [10, 17, 19, 55, 79, 80, 83]. Certainly, we are calling on social science researchers to implement, study, and review innovative and existing IPAC measures to establish their feasibility and utility toward the development of a clear strategy for IPAC within shelter settings. In the meantime, we side with Linder et al. [58] that flexibility with IPAC measures should be considered in shelter care settings to account for their unique differences compared to typical healthcare settings.

This scoping review, along with recent research released by Levesque et al. [102], demonstrated that COVID-19 outbreaks in homeless shelters place an additional burden on frontline workers in this sector—a workforce that is already burnt out and stretched to its limits. One study included in this review [78] reported similar findings to Levesque et al. [102], indicating that staff in the homeless sector are at an increased risk for COVID-19 due in part to the lack of adequate IPAC training and resources available to them. We identified very few documents that focused exclusively on the impacts of COVID-19 among frontline workers in the homeless sector, whereas a significant body of research has been produced about COVID-19 among frontline healthcare workers. This scoping review thus exposes a major gap in the existing literature and signifies a need for future research to investigate the cost and effectiveness of different strategies to protect this population of workers during the present pandemic and future disease outbreaks.

While we call for changes to the homeless shelter system to protect PESH and frontline workers in this sector, the evidence in this scoping review suggests that the best response is preventative. To minimize the risk of infection within homeless shelters, we must eliminate their entry into the shelter system by turning toward homelessness prevention to solve the crisis of homelessness [103]. Several governments worldwide instructed their citizens to “shelter in place” [104] while ignoring the barriers preventing PEH from following such guidance. Thus, federal, provincial/state, and regional governments should strongly consider commitments to implementing two key components of homelessness prevention in the short term: (1) the building of additional affordable housing units to address the shortage of housing experienced worldwide and (2) the removal of barriers to access existing housing for those at risk of or currently experiencing, homelessness [103, 105].

### Strengths and limitations

The content, results, and conclusions of this scoping review should be considered in the context of several strengths and limitations. A systematic search strategy was developed in consultation with a research librarian to include many types of literature sources, databases, pre-print servers, and relevant web pages. The two first authors have extensive knowledge and expertise in homelessness, including lived experience, which removed the need to consult with external experts in the creation of search terms and interpretation of much of the review data. To reduce publication bias in this review, we sought to include gray literature and conducted reference list checks on both academic and gray literature to capture additional relevant sources. A systematic review software was utilized to ensure transparency and accountability of the reviewers during the article selection phase. To minimize selection bias, we ensured two reviewers were independently performing study selection with conflict resolution meetings scheduled with a third reviewer, when necessary, thereby contributing to a high agreement in the process. While caution was taken in article selection, inclusion/exclusion, and inter-screener reliability, we recognize that personal selection bias and subjectivity in interpreting findings and outcomes pose limitations for the themes and conclusions presented in this review.

The researchers used their own logic to set key search terms and did not consult outside experts for the creation of the search terms. Therefore, some key search terms could have been overlooked, which could have produced additional sources of literature. Furthermore, database searches were not exhaustive, and this review is based on very specific temporal parameters for article inclusion (between March 2020 and July 2021). The inclusion of additional databases and an expansion of the period during which the database search and literature collection were conducted could provide additional literature for review. Literature was also restricted to publications in English and higher-income countries, which could be missing additional sources of useful information. An inclusion of lower-income countries would have left this review less focused, and the differences in pandemic response and planning from lower-income countries warrant its own review—which we strongly encourage other researchers to do.

Additionally, given that this is a scoping review, quality assessment was not conducted on each article, as such a critical lens should be applied to the acceptance of the conclusions offered in this review. Importantly, the COVID-19 virus has evolved significantly over the course of the pandemic, with the highly transmissible variants of Delta and Omicron emerging in late 2020 and 2021 [106]. We acknowledge that the timeframe for article sampling was conducted mainly during publications inclusive of the first wave, thus omitting a consideration of the characteristics of the COVID-19 variants and their influence on public health policy. This review samples various communities and nations in a very broad sense which creates issues for consensus and generalizability within and beyond national boundaries. Caution should therefore be taken when utilizing the findings from this study as experiences, outcomes, and responses to homelessness and COVID-19 can differ between communities and nations.

Furthermore, this review did not consider shelters set up specifically for youth or members of LGBTQ2S + communities, in turn excluding a large demographic of the homeless population. Similarly, women may not be represented in the populations of mainstream shelters; therefore, we are limited in the conclusions we can draw about the nature of the relationship between COVID-19 vulnerability, gender, and homelessness [100]. Finally, we were unable to compare patterns in prevalence or IPAC data during specific waves of COVID-19 outbreaks because the onset of each wave of outbreaks varied significantly by geography. It is important then to consider the findings of this scoping review in relation to the adopted temporal, database, and search criteria. As the pandemic continues to progress, newer studies may shift, support, or negate the findings of this review. We invite researchers to replicate or advance the findings presented in this review and leave our findings open to expansion and discussion.

## Conclusion

This scoping review provides evidence that people accessing homeless shelters/hostels and the staff within these facilities are at greater risk of contracting COVID-19 compared to the general population. Asymptomatic spread, existing health, and social vulnerabilities among PEH, capacity issues within the shelter system itself, and limited uptake of recommended IPAC measures are all factors contributing to the high rates of COVID-19 recorded in homeless shelters/hostels in high-income countries.

We suggest urgent transformations to shelter systems that this review helps to inform while calling on researchers to fill the gaps identified in this review as they relate to the feasibility and utility of IPAC strategies tailored to shelter settings. We also reiterate Dej’s [107] argument for moving past reactive crisis-based responses to homelessness and toward homelessness prevention to ultimately prevent entry into the homeless shelter system and reduce the risk of exposure to COVID-19 and other airborne illnesses.

Ultimately, we are at a crossroads where over five million people worldwide have died from COVID-19 and there are dozens of empirical studies, peer-reviewed commentaries, organizational documents, and policy papers providing governments and other decision-makers with the evidence to make sound decisions to prevent more PEH and staff at shelters/hostels from becoming one of those five million. World leaders can either make choices that demonstrate all members of society deserve equitable protection during a global crisis, or they can show that society’s most vulnerable are easily forgotten in times of crisis. We urge all levels of governments in high-income countries to recognize the shortcomings in existing shelter systems that leave PEH vulnerable to damaging health impacts of COVID-19 and other airborne illnesses and allocate substantial resources for meaningful transformations to be made in this sector to protect this vulnerable population now and in the future. 

## Supplementary Information

**Additional file 1.** PRISMA 2020 27-item checklist and abstract checklist.

**Additional file 2.** Articles.

**Additional file 3.** Search of Medline via OVID.

**Additional file 4.** Data extraction tool.

**Additional file 5.** A condensed version of the data repository from all 77 documents.

**Additional file 6.** Definitions of infection control measures.

## Data Availability

The datasets used and/or analyzed during the current study are available from the corresponding author on reasonable request. The authors read and approved the final version of the manuscript.

## Abbreviations

PEH: People experiencing homelessness
PESH: People experiencing sheltered homelessness
PRISMA: Preferred Reporting Items for Systematic Review and Meta-Analysis
PRISMA-ScR: Preferred Reporting Items for Systematic Review and Meta-Analysis Extension for Scoping Reviews
SARS-CoV-2: Severe acute respiratory syndrome coronavirus 2
COVID-19: Coronavirus disease 2019
PCC: Population, concept, and context
IPAC: Infection, prevention, and control
CINAHL: Cumulative Index to Nursing and Allied Health Literature
WHO: World Health Organization
CADTH: Canadian Agency for Drugs and Technologies in Health
CDC: Centers for Disease Control and Prevention
NHCHC: National Health Care for the Homeless Council
PHAC: Public Health Agency of Canada
CNH3: Canadian Network for the Health and Housing of People Experiencing Homelessness
HVAC: Heating, ventilation, and air conditioning
PPE: Personal protective equipment

## References

[1] Balkhair AA (2020). COVID-19 pandemic: a new chapter in the history of infectious diseases. Oman Med J.

[2] World Health Organization (2021). WHO coronavirus (COVID-19) dashboard.

[3] Perri M, Dosani N, Hwang SW (2020). COVID-19 and people experiencing homelessness: challenges and mitigation strategies. CMAJ.

[4] Speak S. The State of Homelessness in Developing Countries [Internet]. Affordable housing and social protection systems for all to address homelessness; 2019; United Nations Office at Nairobi. Available from: https://www.un.org/development/desa/dspd/wp-content/uploads/sites/22/2019/05/SPEAK_Suzanne_Paper.pdf.

[5] The Canadian Observatory on Homelessness (2016). Canadian definition of youth homelessness.

[6] Canadian Observatory on Homelessness. Canadian definition of homelessness [Internet]. Canadian Observatory on Homelessness Press; 2012 . Available from: https://www.homelesshub.ca/resource/canadian-definition-homelessness Cited 25 Oct 2021

[7] Lima NNR, de Souza RI, Feitosa PWG, de Sousa Moreira JL, da Silva CGL, Neto MLR (2020). People experiencing homelessness: their potential exposure to COVID-19. Psychiatry Res.

[8] Imbert E, Kinley PM, Scarborough A, Cawley C, Sankaran M, Cox SN, et al. Coronavirus Disease 2019 Outbreak in a San Francisco Homeless Shelter. Clin Infect Dis. 2021;73(2):324–7. 10.1093/cid/ciaa1071 PMC7454344 32744615

[9] Richard L, Booth R, Rayner J, Clemens KK, Forchuk C, Shariff SZ (2021). Testing, infection and complication rates of COVID-19 among people with a recent history of homelessness in Ontario, Canada: a retrospective cohort study. CMAJ Open.

[10] Loubiere S, Monfardini E, Allaria C, Mosnier M, Allibert A, Ninove L, et al. Seroprevalence of SARS-CoV-2 antibodies among homeless people living rough, in shelters and squats: A large population-based study in France. PLoS One. 2021;16(9):e0255498. 10.1371/journal.pone.0255498 PMC8443066 34525096

[11] Kiran T, Craig-Neil A, Das P, Lockwood J, Wang R, Nathanielsz N (2021). Factors associated with SARS-CoV-2 positivity in 20 homeless shelters in Toronto, Canada, from April to July 2020: a repeated cross-sectional study. CMAJ Open.

[12] Baggett TP, Keyes H, Sporn N, Gaeta JM (2020). Prevalence of SARS-CoV-2 infection in residents of a large homeless shelter in Boston. JAMA.

[13] Ralli M, Morrone A, Arcangeli A, Ercoli L (2021). Asymptomatic patients as a source of transmission of COVID-19 in homeless shelters. Int J Infect Dis.

[14] Lewer D, Braithwaite I, Bullock M, Eyre MT, White PJ, Aldridge RW (2020). COVID-19 among people experiencing homelessness in England: a modelling study. Lancet Respir Med.

[15] Mosites E, Parker E, Clarke K, Gaeta J, Baggett T, Imbert E (2020). Assessment of SARS-COV-2 infection prevalence in homeless shelters - four U.S. Cities, March 27–April 15, 2020. MMWR Morb Mortal Wkly Rep.

[16] Ly TDA, Hoang VT, Goumballa N, Louni M, Canard N, Dao TL (2021). Variations in respiratory pathogen carriage among a homeless population in a shelter for men in Marseille, France, March-July 2020: cross-sectional 1-day surveys. Eur J Clin Microbiol Infect Dis.

[17] Chapman LAC, Kushel M, Cox SN, Scarborough A, Cawley C, Nguyen TQ (2021). Comparison of infection control strategies to reduce COVID-19 outbreaks in homeless shelters in the United States: a simulation study. BMC Med.

[18] Centers for Disease Control and Prevention. Snapshot of CDC guidance for homeless and meal service providers for emergency and day shelters [Internet]. Centers for Disease Control and Prevention; 2020 . Available from: https://www.cdc.gov/coronavirus/2019-ncov/community/homeless-shelters/homeless-service-provider-guidance.html Cited 15 Sep 2021

[19] Mohsenpour A, Bozorgmehr K, Rohleder S, Stratil J, Costa D (2021). SARS-Cov-2 prevalence, transmission, health-related outcomes and control strategies in homeless shelters: systematic review and meta-analysis. EClinicalMedicine.

[20] Fuchs JD, Carter HC, Evans J, Graham-Squire D, Imbert E, Bloome J (2021). Assessment of a hotel-based COVID-19 isolation and quarantine strategy for persons experiencing homelessness. JAMA Netw Open.

[21] Arksey H, O’Malley L (2005). Scoping studies: towards a methodological framework. Int J Soc Res Methodol.

[22] Levac D, Colquhoun H, O’Brien KK (2010). Scoping studies: advancing the methodology. Implement Sci.

[23] Peters MD, Godfrey C, Mcinerney P, Munn Z, Tricco AC, Khalil H, Aromataris E, Munn Z (2020). Chapter 11: scoping reviews. BI Manual for Evidence Synthesis.

[24] Tricco AC, Lillie E, Zarin W, O’Brien KK, Colquhoun H, Levac D (2018). PRISMA extension for scoping reviews (PRISMA-ScR): checklist and explanation. Ann Intern Med.

[25] Tenny S, Hoffman MR. Prevalence [Internet]. StatPearls [Internet]. StatPearls Publishing; 2021 . Available from: https://www.ncbi.nlm.nih.gov/books/NBK430867/ Cited 26 Jun 2022

[26] Jadidzadeh A, Kneebone R (2020). Homeless shelter flows in calgary and the potential impact of COVID-19. Can Public Policy.

[27] Page MJ, McKenzie JE, Bossuyt PM, Boutron I, Hoffmann TC, Mulrow CD (2021). The PRISMA 2020 statement: an updated guideline for reporting systematic reviews. BMJ.

[28] Aranda-Díaz A, Imbert E, Strieff S, Graham-Squire D, Evans JL, Moore J, et al. Implementation of Rapid and Frequent SARS-CoV2 Antigen Testing and Response in Congregate Homeless Shelters [Internet]. medRxiv; 2021 [cited 2022 Oct 13]. Available from: https://www.medrxiv.org/content/10.1101/2021.04.20.21255787v1. 10.1371/journal.pone.0264929 PMC8912252 35271622

[29] Arroyo L (2020). Homeless service provider interim COVID-19 response plan 202.

[30] Baggett TP, Keyes H, Sporn N, Gaeta JM. COVID-19 outbreak at a large homeless shelter in Boston: Implications for universal testing [Internet]. medRxiv; 2020 [cited 2022 Oct 13]. Available from: https://www.medrxiv.org/content/10.1101/2020.04.12.20059618v1.

[31] Baggett TP, Racine MW, Lewis E, De Las ND, O’Connell JJ, Bock B (2020). Addressing COVID-19 among people experiencing homelessness: description, adaptation, and early findings of a multiagency response in Boston. Public Health Rep.

[32] Baggett TP, Scott JA, Le MH, Shebl FM, Panella C, Losina E (2020). Clinical outcomes, costs, and cost-effectiveness of strategies for adults experiencing sheltered homelessness during the COVID-19 pandemic. JAMA Netw Open.

[33] Baltimore City Health Department (2020). Baltimore City COVID-19 congregate homeless shelter response.

[34] Baral S, Bond A, Boozary A, Bruketa E, Elmi N, Freiheit D (2021). Seeking shelter: homelessness and COVID-19.

[35] Barocas JA, Jacobson KR, Hamer DH (2021). Addressing the COVID-19 pandemic among persons experiencing homelessness: steps to protect a vulnerable population. J Gen Intern Med.

[36] Bond A (2020). COVID-19 response framework for people experiencing homelessness.

[37] California Business Consumer Services and Housing Agency. Interim guidance for homeless assistance providers on novel coronavirus (COVID-19) [Internet]. California; 2020 . Available from: https://bcsh.ca.gov/hcfc/documents/covid19_interim_guidance.pdf Cited 15 Sep 2021

[38] Canadian Network for the Health and Housing of People Experiencing Homelessness (2020). Briefing and recommendations: isolation and quarantine COVID-19 in the homelessness service sector.

[39] Center on Budget and Policy Priorities, National Alliance to End Homelessness, National Low Income Housing Coalition, National Health Care for the Homeless Council. The framework for an equitable COVID-19 homelessness response [Internet]. 2020 Oct [cited 2021 Sep 15]. Available from: https://www.homelesshub.ca/resource/framework-equitable-covid-19-homelessness-response

[40] Centers for Disease Control and Prevention. Interim guidance for homeless service providers to plan and respond to coronavirus disease 2019 (COVID-19) [Internet]. USA: Centers for Disease Control and Prevention; 2020 Apr p. 7. Available from: https://www.cdc.gov/coronavirus/2019-ncov/downloads/COVID19_Homeless-H.pdf

[41] CDC (2020). Interim guidance for SARS-CoV-2 testing in homeless shelters and encampments.

[42] Chicago Homelessness and Health Response Group For Equity. Building an effective cross-sector partnership to address COVID-19 among vulnerably housed populations of Chicago [Internet]. Chicago Homelessness and Health Response Group for Equity; 2020 Nov [cited 2021 Sep 15]. Available from: https://regroup-production.s3.amazonaws.com/documents/ReviewReference/359498609/CHHRGE-White-Paper-06.11.2020_1.pdf?AWSAccessKeyId=AKIAJBZQODCMKJA4H7DA&Expires=1631733582&Signature=BD18mE9emTBH%2F6Nwas5c6nGIzrI%3D

[43] Department of Homeless Services (2020). COVID-19 isolation plan and best practices for DHS shelters.

[44] Department of Homeless Services (2020). COVID-19 best practices for DHS shelters.

[45] Department of Homeless Services (2020). DHS COVID-19 isolation site process, responsibilities, and best practices.

[46] Department of Public Health (2021). Los Angeles County Department of Public Health: guidance for homeless shelters.

[47] Falvo N. Isolation, Physical Distancing and Next Steps Regarding Homelessness: A Scan of 12 Canadian Cities [Internet]. Calgary, Alberta: The Homeless Hub; 2020 Dec [cited 2021 Sep 15] p. 54. Available from: https://www.calgaryhomeless.com/wp-content/uploads/2020/12/Isolation-Physical-Distancing-and-Next-Steps-Regarding-Homelessness_2020_12_071.pdf.

[48] Gewirtz O’Brien JR, Auerswald C, English A, Ammerman S, Beharry M, Heerde JA (2021). Youth experiencing homelessness during the COVID-19 pandemic: unique needs and practical strategies from international perspectives. J Adolesc Health.

[49] Ghinai I, Davis ES, Mayer S, Toews KA, Huggett TD, Snow-Hill N (2020). Risk factors for severe acute respiratory syndrome coronavirus 2 infection in homeless shelters in Chicago, Illinois-March-May, 2020. Open Forum Infect Dis.

[50] Government of New Brunswick (2020). COVID-19 guidance for homeless shelters.

[51] Government of New Brunswick. New Brunswick Public Health COVID-19 Guidance: for persons working with individuals experiencing housing instability [Internet]. New Brunswick: Department of Health Public Health New Brunswick; 2021 Feb [cited 2021 Sep 15]. Available from: https://www2.gnb.ca/content/dam/gnb/Departments/h-s/pdf/Individuals-Experiencing-Housing-Instability.pdf

[52] Government of Northwest Territories (2020). NWT guidance for providers of services to individuals experiencing homelessness.

[53] Government of Yukon (2020). Guidance for the prevention and management of COVID-19 in communal living settings.

[54] Jameson A, Curtis K, Pelkey L, Lynn L, Bora N, Jacques L. Widescale SARS-Cov-2 Testing in Individuals Experiencing Homelessness in a Medium-Sized Midwest City. J Med Public Health. 2021;2(1014):2.

[55] Karb R, Samuels E, Vanjani R, Trimbur C, Napoli A (2020). Homeless shelter characteristics and prevalence of SARS-CoV-2. West J Emerg Med.

[56] Kelly D, Murphy H, Vadlamudi R, Kraut R, Dalessio K, Malani AN (2020). Successful public health measures preventing coronavirus disease 2019 (COVID-19) at a Michigan homeless shelter. Infect Control Hosp Epidemiol.

[57] Kiran T, Craig-Neil A, Das P, Lockwood J, Wang R, Nathanielsz N, et al. Mobile outreach testing for COVID-19 in twenty homeless shelters in Toronto, Canada [Internet]. medRxiv; 2020 [cited 2022 Oct 13]. Available from: https://www.medrxiv.org/content/10.1101/2020.11.23.20235465v1.

[58] Lindner AK, Sarma N, Rust LM, Hellmund T, Krasovski-Nikiforovs S, Wintel M, et al. Monitoring for COVID-19 by universal testing in a homeless shelter in Germany: a prospective feasibility cohort study. BMC Infect Dis. 2021;21(1):1241. 10.1186/s12879-021-06945-4 PMC8665323 34895157

[59] London Coronavirus Response Cell (2021). Frequently asked questions (FAQs) for hostels, and homeless hotels when dealing with COVID-19.

[60] Ly TDA, Nguyen NN, Hoang VT, Goumballa N, Louni M, Canard N (2021). Screening of SARS-CoV-2 among homeless people, asylum-seekers and other people living in precarious conditions in Marseille, France, March-April 2020. Int J Infect Dis.

[61] Mercy Care (2020). Universal COVID-19 testing for people experiencing homelessness.

[62] Ministry of Health. COVID-19 guidance: homeless shelters [Internet]. Ontario: Ministry of Health; 2020 Apr p. 10. Available from: https://www.health.gov.on.ca/en/pro/programs/publichealth/coronavirus/docs/2019_homeless_shelters_guidance.pdf

[63] Monroe County Shelter Bed Task Group (2020). Guidance to shelter providers and other community partners in operating under COVID-19 requirements.

[64] S Mosquera-Bruno (2020). Preparing shelters for COVID-19.

[65] Murray C (2021). COVID-19 response update: protecting people experiencing homelessness and ensuring the safety of the shelter system.

[66] National Alliance to End Homelessness. Homelessness & COVID-19: considerations and action steps. 2020 Mar [cited 2021 Sep 15]; Available from: https://endhomelessness.org/resource/homelessness-covid-19-considerations-and-action-steps/

[67] National Collaborating Centre for Methods and Tools. Rapid review update 1: what factors increase the risk of COVID-19 outbreaks in congregate living settings? How do outcomes compare to outbreaks in community settings? [Internet]. Hamilton, Ontario: National Collaborating Centre for Methods and Tools; 2020 Jul [cited 2021 Sep 15]. Available from: https://www.nccmt.ca/covid-19/covid-19-rapid-evidence-service/17

[68] National Health Care for the Homeless Counci (2020). Needed actions from public health and emergency response systems.

[69] North Carolina Department of Health and Human Services (2020). Interim coronavirus disease 2019 (COVID-19) guidance for homeless shelters and service providers.

[70] NYC Department of Health and Mental Hygiene (2020). Interim COVID-19 guidance for homeless shelters.

[71] O’Shea T, Bodkin C, Mokashi V, Beal K, Wiwcharuk J, Lennox R (2021). Pandemic planning in homeless shelters: a pilot study of a coronavirus disease 2019 (COVID-19) testing and support program to mitigate the risk of COVID-19 outbreaks in congregate settings. Clin Infect Dis.

[72] Office of Temporary and Disability Assistance (2020). Interim guidance for operators of congregate facilities providing shelter to individuals who are homeless.

[73] Public Health Agency of Canada (2020). Guidance for providers of services for people experiencing homelessness (in the context of COVID-19).

[74] Public Health England (2021). COVID-19: guidance for commissioners and providers of hostel services for people experiencing homelessness and rough sleeping.

[75] Public Health Ontario. Health protection actions for people experiencing homelessness during the COVID-19 pandemic [Internet]. Toronto: Public Health Ontario; 2021 Mar p. 16. Available from: https://www.publichealthontario.ca/-/media/documents/ncov/he/2021/02/covid-19-homelessness-environmental-scan.pdf?sc_lang=en

[76] Ralli M, Arcangeli A, Ercoli L (2021). Homelessness and COVID-19: leaving no one behind. Ann Glob Health.

[77] Ralli M, Arcangeli A, Morrone A, Ercoli L (2021). Homeless shelter characteristics and prevalence of SARS-CoV-2. West J Emerg Med.

[78] Rao CY, Robinson T, Huster K, Laws RL, Keating R, Tobolowsky FA, et al. Occupational exposures and mitigation strategies among homeless shelter workers at risk of COVID-19. PLoS One. 2021;16(11):e0253108. 10.1371/journal.pone.0253108 PMC8559982 34723986

[79] Redditt V, Wright V, Rashid M, Male R, Bogoch I (2020). Outbreak of SARS-CoV-2 infection at a large refugee shelter in Toronto, April 2020: a clinical and epidemiologic descriptive analysis. CMAJ Open.

[80] Roederer T, Mollo B, Vincent C, Nikolay B, Llosa AE, Nesbitt R (2021). Seroprevalence and risk factors of exposure to COVID-19 in homeless people in Paris, France: a cross-sectional study. The Lancet Public Health.

[81] Rogers JH, Link AC, McCulloch D, Brandstetter E, Newman KL, Jackson ML (2021). Characteristics of COVID-19 in homeless shelters : a community-based surveillance study. Ann Intern Med.

[82] Roland M, Abdelhafidh LB, Déom V, Vanbiervliet F, Coppieters Y, Racapé J (2021). SARS-CoV-2 screening among people living in homeless shelters in Brussels, Belgium. PLoS ONE.

[83] Samuels EA, Karb R, Vanjani R, Trimbur MC, Napoli A. Congregate Shelter Characteristics and Prevalence of Asymptomatic SARS-CoV-2 [Internet]. medRxiv; 2020 [cited 2022 Oct 13]. Available from: https://www.medrxiv.org/content/10.1101/2020.05.21.20108985v2. 10.5811/westjem.2020.7.48725 PMC7514394 32970553

[84] Self JL, Montgomery MP, Toews KA, Samuels EA, Imbert E, McMichael TM (2021). Shelter characteristics, infection prevention practices, and universal testing for SARS-CoV-2 at homeless shelters in 7 US urban areas. Am J Public Health.

[85] Tobolowsky FA. COVID-19 outbreak among three affiliated homeless service sites—King County, Washington, 2020. MMWR Morb Mortal Wkly Rep [Internet]. 2020;69. Available from: https://www.cdc.gov/mmwr/volumes/69/wr/mm6917e2.htm Cited 15 Sep 2021 10.15585/mmwr.mm6917e2 PMC7206987 32352954

[86] Udechukwu T, Harnett L, Mabhala M, Reid J. COVID-19 and people experiencing homelessness: reported measures implemented in the European Region during the Pandemic. Associations of the Schools of Public Health European Region; 2021 Apr.

[87] Vancouver Coastal Health (2020). Operations manual for temporary COVID-19 shelter for vulnerable populations in vancouver coastal health.

[88] Wang L, Ma H, Yiu KCY, Calzavara A, Landsman D, Luong L, et al. Heterogeneity in testing, diagnosis and outcome in SARS-CoV-2 infection across outbreak settings in the Greater Toronto Area, Canada: an observational study. CMAJ Open. 2020;8(4):E627-36. 10.9778/cmajo.20200213 PMC7567509 33037070

[89] Wang J, Majumder M, Johnson C (2020). Policy brief: policies protecting people experiencing homelessness during the COVID-19 pandemic.

[90] Yoon JC, Montgomery MP, Buff AM, Boyd AT, Jamison C, Hernandez A, et al. COVID-19 prevalence among people experiencing homelessness and homelessness service staff during early community transmission in Atlanta, Georgia, April-May 2020. Clin Infect Dis 2020;ciaa1340. 10.1093/cid/ciaa1340 PMC7499502 32898272

[91] Department of Housing and Urban Development (2020). Shelter management during an infectious disease outbreak.

[92] Steer KJD, Klassen DC, O’Gorman CM, Webster M, Mitchell M, Krichevsky L (2021). Cups for COVID: rapid implementation of a harm reduction initiative to support populations experiencing homelessness during the COVID-19 pandemic. Can J Public Health.

[93] Department of Housing and Urban Development (2020). Homeless system response: COVID-19 preparedness checklist for shelter facilities.

[94] Gaetz S, Dej E, Richter T, Redman M (2016). The state of homelessness in Canada 2016.

[95] Fazel S, Geddes JR, Kushel M (2014). The health of homeless people in high-income countries: descriptive epidemiology, health consequences, and clinical and policy recommendations. The Lancet.

[96] Ministry of Housing, Communities & Local Government. Statutory homelessness in England: October to December 2021 [Internet]. 2022 [cited 2022 Jul 10]. Available from: https://www.gov.uk/government/statistics/statutory-homelessness-in-england-october-to-december-2021

[97] Department for Levelling Up, Housing and Communities, Ministry of Housing, Communities & Local Government. Live tables on homelessness [Internet]. 2022 [cited 2022 Jul 10]. Available from: https://www.gov.uk/government/statistical-data-sets/live-tables-on-homelessness

[98] The United States Department of Housing and Urban Development (2022). 2021 Annual Homeless Assessment Report (AHAR) to congress: part 1 point in time estimates of sheltered homelessness.

[99] Employment and Social Development Canada. Shelter Capacity Report 2019 [Internet]. Canada; 2021 Jun [cited 2022 Jul 10]. Available from: https://www.canada.ca/en/employment-social-development/employment-social-development/homelessness/publications-bulletins/shelter-capacity-2019.html

[100] Schwan K, Versteegh A, Perri M, Caplan R, Baig K, Dej E, et al. The state of women’s housing need & homelessness in Canada [Internet]. Toronto, Canada: Canadian Observatory on Homelessness Press; 2020. Available from: https://womenshomelessness.ca/literature-review/

[101] Moffa M, Cronk R, Fejfar D, Dancausse S, Padilla LA, Bartram J (2019). A systematic scoping review of environmental health conditions and hygiene behaviors in homeless shelters. Int J Hyg Environ Health.

[102] Levesque J, Sehn C, Babando J, Ecker J, Embleton L (2021). Understanding the needs of workers in the homelessness and housing sector: final report.

[103] Gaetz S, Dej E (2017). A new direction: a framework for homelessness prevention.

[104] Nast C (2020). How do you shelter in place when you don’t have a home? The New Yorker.

[105] Pleace N (2019). Preventing homelessness: a review of the international evidence.

[106] Tian D, Sun Y, Xu H, Ye Q (2022). The emergence and epidemic characteristics of the highly mutated SARS-CoV-2 Omicron variant. J Med Virol.

[107] Dej E, Gaetz S, Schwan K (2020). Turning off the tap: a typology for homelessness prevention. J Primary Prevent.

