# Supplementary material for: COVID-19 prevalence and infection control measures at homeless shelters and hostels in high-income countries: a scoping review
Source: Syst Rev. 2022 Oct 15;11:223. doi: 10.1186/s13643-022-02089-x (PMC9569412; doi:10.1186/s13643-022-02089-x)
Supplement: Supplementary file 3 — Additional file 3. Search of Medline via OVID. [file 13643_2022_2089_MOESM3_ESM.docx]

Supplementary File C

Search of Medline via OVID

| Search | Boolean Operators | Keywords |
| --- | --- | --- |
| 1 |  | (Homeless persons or Homeless people or Homeless youth or Homeless adults or People experiencing homelessness or Homelessness).mp. [mp=title, abstract, original title, name of substance word, subject heading word, floating sub-heading word, keyword heading word, organism supplementary concept word, protocol supplementary concept word, rare disease supplementary concept word, unique identifier, synonyms] |
| 2 | AND | (COVID-19 or SARS-CoV-2 or Coronavirus or Coronavirus Disease or Coronavirus Disease 2019).mp. [mp=title, abstract, original title, name of substance word, subject heading word, floating sub-heading word, keyword heading word, organism supplementary concept word, protocol supplementary concept word, rare disease supplementary concept word, unique identifier, synonyms] |
| 3 | AND | (Homeless shelter or Emergency shelter or Homeless hostel or Hostel or Warming center or Family violence shelter).mp. [mp=title, abstract, original title, name of substance word, subject heading word, floating sub-heading word, keyword heading word, organism supplementary concept word, protocol supplementary concept word, rare disease supplementary concept word, unique identifier, synonyms] |
| 4 | 1 AND 2 AND 3 | (Homeless persons or Homeless people or Homeless youth or Homeless adults or People experiencing homelessness or Homelessness).mp. [mp=title, abstract, original title, name of substance word, subject heading word, floating sub-heading word, keyword heading word, organism supplementary concept word, protocol supplementary concept word, rare disease supplementary concept word, unique identifier, synonyms] AND (COVID-19 or SARS-CoV-2 or Coronavirus or Coronavirus Disease or Coronavirus Disease 2019).mp. [mp=title, abstract, original title, name of substance word, subject heading word, floating sub-heading word, keyword heading word, organism supplementary concept word, protocol supplementary concept word, rare disease supplementary concept word, unique identifier, synonyms] AND (Homeless shelter or Emergency shelter or Homeless hostel or Hostel or Warming center or Family violence shelter).mp. [mp=title, abstract, original title, name of substance word, subject heading word, floating sub-heading word, keyword heading word, organism supplementary concept word, protocol supplementary concept word, rare disease supplementary concept word, unique identifier, synonyms] |
| 5 | 1 OR 3 | (Homeless persons or Homeless people or Homeless youth or Homeless adults or People experiencing homelessness or Homelessness).mp. [mp=title, abstract, original title, name of substance word, subject heading word, floating sub-heading word, keyword heading word, organism supplementary concept word, protocol supplementary concept word, rare disease supplementary concept word, unique identifier, synonyms] OR (Homeless shelter or Emergency shelter or Homeless hostel or Hostel or Warming center or Family violence shelter).mp. [mp=title, abstract, original title, name of substance word, subject heading word, floating sub-heading word, keyword heading word, organism supplementary concept word, protocol supplementary concept word, rare disease supplementary concept word, unique identifier, synonyms] |
| 6 | 2 AND 5 | (COVID-19 or SARS-CoV-2 or Coronavirus or Coronavirus Disease or Coronavirus Disease 2019).mp. [mp=title, abstract, original title, name of substance word, subject heading word, floating sub-heading word, keyword heading word, organism supplementary concept word, protocol supplementary concept word, rare disease supplementary concept word, unique identifier, synonyms] AND (Homeless persons or Homeless people or Homeless youth or Homeless adults or People experiencing homelessness or Homelessness).mp. [mp=title, abstract, original title, name of substance word, subject heading word, floating sub-heading word, keyword heading word, organism supplementary concept word, protocol supplementary concept word, rare disease supplementary concept word, unique identifier, synonyms] OR (Homeless shelter or Emergency shelter or Homeless hostel or Hostel or Warming center or Family violence shelter).mp. [mp=title, abstract, original title, name of substance word, subject heading word, floating sub-heading word, keyword heading word, organism supplementary concept word, protocol supplementary concept word, rare disease supplementary concept word, unique identifier, synonyms] |

*No other restrictions placed on search

**Note Ovid through York University Libraries is set to automatically “Map Term to Subject Heading”. Ensure this option is selected.
