# Supplementary material for: COVID-19 prevalence and infection control measures at homeless shelters and hostels in high-income countries: a scoping review
Source: Syst Rev. 2022 Oct 15;11:223. doi: 10.1186/s13643-022-02089-x (PMC9569412; doi:10.1186/s13643-022-02089-x)
Supplement: Supplementary file 6 — Additional file 6. Definitions of infection control measures. [file 13643_2022_2089_MOESM6_ESM.docx]

**Definitions of Infection Control Measures**

Screening/Surveillance: Screening refers to a series of questions asked to determine a person's risk for COVID-19 (e.g., experience of symptoms, recent travel, pre-existing conditions) (1). Surveillance refers to other steps taken for ongoing systematic collection, analysis, and interpretation of health-related data (e.g., sentinel surveillance in shelters) (2).

Testing: A viral or antibody test to determine if an individual currently or previously was infected with COVID-19 (3).

Hand and Respiratory Etiquette: Refers to any actions taken to reduce the spread of pathogens from hands or through the air (e.g., hand washing, hand sanitizer, and covering cough or sneeze) (4,5).

Personal Protective Equipment: Any clothing or material donned by workers to protect from hazards (5).

Environmental Cleaning and Waste Management: Environmental cleaning refers to consistent and proper cleaning/disinfection of surfaces and equipment that could become contaminated (5). Waste management refers to the appropriate and safe disposal of waste products (5).

Physical Distancing: Ensuring a distance of 2 metres or 6 feet is maintained between people and activities in shelters are limited (6).

Isolation and Quarantine: Isolation involves separating people infected with COVID-19 from those who are not infected and quarantine refers to keeping those who have been in close contact with people who have COVID-19 away from others (7).

Food Safety: Any measures taken related to serving, preparing, storing, and eating food in shelters/hostels which applies to food being prepared in shelters or delivered to these facilities (8).

Other: any other measures taken to prevent the spread of COVID-19 that are not captured by the previous eight categories (e.g., expanding shelter hours, enhancing ventilation).

**References**

1. Government of Ontario. Screening for COVID-19: guidance for employers [Internet]. ontario.ca. 2021 [cited 2021 Nov 25]. Available from: http://www.ontario.ca/page/screening-covid-19-guidance-employers

2. Oleske DM. Screening and Surveillance for Promoting Population Health. Epidemiology and the Delivery of Health Care Services. 2009 Apr 2;131–50.

3. Centers for Disease Control and Prevention. COVID-19 Testing Overview [Internet]. 2020 [cited 2021 Nov 25]. Available from: https://www.cdc.gov/coronavirus/2019-ncov/symptoms-testing/testing.html

4. Oregon Department of Human Services. Hand and Respiratory Hygiene [Internet]. 2017 [cited 2021 Nov 25]. Available from: https://www.oregon.gov/DHS/SENIORS-DISABILITIES/PROVIDERS-PARTNERS/Documents/0724B-Hand-and-Respiratory-Hygiene-Modified.pdf

5. Public Health Ontario. Best Practices for Environmental Cleaning for Prevention and Control of Infections in All Health Care Settings [Internet]. Ontario; 20118 [cited 2021 Nov 25]. Report No.: 3. Available from: https://www.publichealthontario.ca/-/media/documents/B/2018/bp-environmental-cleaning.pdf

6. Janovsky S. COVID-19: Physical Distancing. 2020.

7. Centers for Disease Control and Prevention. COVID-19 Quarantine vs. Isolation. 2021.

8. IPAC Canada. Infection Prevention and Control (IPAC) Program Standard [Internet]. [cited 2021 Nov 25]. Available from: http://ipac-canada.org/photos/custom/pdf/IPAC_PROGRAM_STANDARD_2016.pdf
